# Supplementary material for: DNA barcoding for the assessment of marine and coastal fish diversity from the Coast of Mozambique
Source: PLoS One. 2024 Feb 6;19(2):e0293345. doi: 10.1371/journal.pone.0293345 (PMC10846724; doi:10.1371/journal.pone.0293345)
Supplement: S2 Table — The distribution of sequence divergence at each taxonomic level based on the Pairwise Distance model. (DOCX) [file pone.0293345.s002.docx]

| **OTU** | **Mean** | **Max** | **Count** | **NN Dist** |
| --- | --- | --- | --- | --- |
| OTU-1 | 0.0 | 0.0 | 4 | 11.437.908 |
| OTU-2 | 0.1362862 | 0.3407155 | 5 | 8.143.322 |
| OTU-3 | 0.27649698 | 0.5681818 | 4 | 8.143.322 |
| OTU-4 | 14.897.579 | 14.897.579 | 2 | 16.945.997 |
| OTU-5 | 0 | 0 | 1 | 16.091.955 |
| OTU-6 | 0.19379845 | 0.19379845 | 2 | 11.018.364 |
| OTU-7 | 0.0 | 0.0 | 3 | 13.942.307 |
| OTU-8 | 0.0 | 0.0 | 3 | 69.892.473 |
| OTU-9 | 0.20219055 | 0.5050505 | 5 | 69.892.473 |
| OTU-10 | 0.0 | 0.0 | 4 | 13.976.706 |
| OTU-11 | 0.0 | 0.0 | 5 | 17.307.693 |
| OTU-12 | 0.13300274 | 0.23640662 | 3 | 40.518.637 |
| OTU-13 | 0 | 0 | 1 | 40.518.637 |
| OTU-14 | 0.0 | 0.0 | 2 | 86.046.505 |
| OTU-15 | 0.0 | 0.0 | 4 | 9.397.163 |
| OTU-16 | 0.42057228 | 0.6472492 | 5 | 5.365.854 |
| OTU-17 | 0.10726798 | 0.16129032 | 3 | 5.365.854 |
| OTU-18 | 0 | 0 | 1 | 14.455.782 |
| OTU-19 | 13.215.859 | 13.215.859 | 2 | 10.489.511 |
| OTU-20 | 0 | 0 | 1 | 164.076 |
| OTU-21 | 0.5102041 | 0.5102041 | 2 | 14.455.782 |
| OTU-22 | 0.0 | 0.0 | 2 | 72.769.957 |
| OTU-23 | 0.27446485 | 0.44052863 | 3 | 1.659.919 |
| OTU-24 | 0 | 0 | 1 | 8.493.589 |
| OTU-25 | 0.1083611 | 0.16366613 | 4 | 17.932.148 |
| OTU-26 | 0.32051283 | 0.48076925 | 3 | 107.081.175 |
| OTU-27 | 0 | 0 | 1 | 8.493.589 |
| OTU-28 | 0.10822625 | 0.16286644 | 4 | 17.723.577 |
| OTU-29 | 0.13111888 | 0.23310024 | 4 | 18.589.745 |
| OTU-30 | 0.120623104 | 0.20161289 | 3 | 1.474.359 |
| OTU-31 | 0 | 0 | 1 | 18.609.024 |
| OTU-32 | 0 | 0 | 1 | 20.050.125 |
| OTU-33 | 0.21970783 | 0.49668875 | 4 | 11.952.862 |
| OTU-34 | 0.21367522 | 0.32051283 | 3 | 9.777.016 |
| OTU-35 | 0 | 0 | 1 | 12.841.092 |
| OTU-36 | 0.23364486 | 0.23364486 | 2 | 14.018.692 |
| OTU-37 | 0 | 0 | 1 | 11.631.664 |
| OTU-38 | 0.4854369 | 0.4854369 | 2 | 14.102.565 |
| OTU-39 | 0.17331475 | 0.35211268 | 4 | 13.043.478 |
| OTU-40 | 0 | 0 | 1 | 11.631.664 |
| OTU-41 | 0.09623102 | 0.16051364 | 5 | 35.256.412 |
| OTU-42 | 0.21436448 | 0.32258064 | 4 | 35.256.412 |
| OTU-43 | 0.4878049 | 0.4878049 | 2 | 14.308.943 |
| OTU-44 | 0 | 0 | 1 | 17.014.446 |
| OTU-45 | 0.14259967 | 0.37243947 | 5 | 15.865.384 |
| OTU-46 | 0 | 0 | 1 | 13.646.056 |
| OTU-47 | 0.0 | 0.0 | 2 | 77.083.335 |
| OTU-48 | 0.107619345 | 0.16260162 | 3 | 156.928.215 |
| OTU-49 | 0 | 0 | 1 | 12.195.122 |
| OTU-50 | 0.13663477 | 0.2247191 | 3 | 1.574.074 |
| OTU-51 | 0 | 0 | 1 | 1.574.074 |
| OTU-52 | 0.32786885 | 0.4918033 | 3 | 19.344.263 |
| OTU-53 | 0 | 0 | 1 | 9.150.327 |
| OTU-54 | 0 | 0 | 1 | 9.150.327 |
| OTU-55 | 0.38461536 | 0.8012821 | 5 | 13.621.796 |
| OTU-56 | 0.21930565 | 0.48309177 | 3 | 16.869.566 |
| OTU-57 | 0.32102728 | 0.32102728 | 2 | 77.083.335 |
| OTU-58 | 0 | 0 | 1 | 16.476.347 |
| OTU-59 | 0.0 | 0.0 | 2 | 15.845.825 |
| OTU-60 | 0.9615385 | 14.423.077 | 3 | 11.917.099 |
| OTU-61 | 0.0 | 0.0 | 3 | 9.455.128 |
| OTU-62 | 0.16941716 | 0.33613446 | 3 | 14.453.782 |
| OTU-63 | 0 | 0 | 1 | 9.455.128 |
| OTU-64 | 0 | 0 | 1 | 12.704.918 |
| OTU-65 | 0 | 0 | 1 | 12.179.487 |
| OTU-66 | 0.10683761 | 0.16025642 | 4 | 9.455.128 |
| OTU-67 | 0.0 | 0.0 | 3 | 9.455.128 |
| OTU-68 | 0.08012821 | 0.16025642 | 4 | 12.660.256 |
| OTU-69 | 0 | 0 | 1 | 18.108.974 |
| OTU-70 | 0 | 0 | 1 | 11.055.277 |
| OTU-71 | 0.642574 | 0.9615385 | 3 | 11.378.204 |
| OTU-72 | 0 | 0 | 1 | 9.777.016 |
| OTU-73 | 0 | 0 | 1 | 72.769.957 |
| OTU-74 | 0.5319149 | 0.5319149 | 2 | 1.897.163 |
| OTU-75 | 0 | 0 | 1 | 15.384.616 |
| OTU-76 | 0.14993119 | 0.3629764 | 4 | 11.648.746 |
| OTU-77 | 0.14098419 | 0.3552398 | 5 | 10.256.411 |
| OTU-78 | 0.24653761 | 0.5235602 | 5 | 15.116.279 |
| OTU-79 | 0.064154096 | 0.1607717 | 5 | 1.745.283 |
| OTU-80 | 0.14177164 | 0.34013605 | 4 | 15.816.326 |
| OTU-81 | 0.3469224 | 0.5208334 | 3 | 107.081.175 |
| OTU-82 | 0 | 0 | 1 | 14.634.146 |
| OTU-83 | 0.17462178 | 0.34965035 | 4 | 11.519.198 |
| OTU-84 | 0.6730769 | 11.217.949 | 5 | 36.858.976 |
| OTU-85 | 0.085910656 | 0.17182131 | 4 | 5.448.718 |
| OTU-86 | 0.29623872 | 0.5494506 | 5 | 16.049.381 |
| OTU-87 | 0.0 | 0.0 | 2 | 36.858.976 |
| OTU-88 | 0 | 0 | 1 | 15.337.424 |
| OTU-89 | 0.0 | 0.0 | 2 | 12.660.256 |
| OTU-90 | 0.3552398 | 0.3552398 | 2 | 6.25 |
| OTU-91 | 0.0 | 0.0 | 3 | 6.25 |
| OTU-92 | 0.08056194 | 0.16260162 | 4 | 17.467.949 |
| OTU-93 | 0.0 | 0.0 | 4 | 14.067.796 |
| OTU-94 | 0.22875819 | 0.4901961 | 5 | 16.987.179 |
| OTU-95 | 0.27022097 | 0.32520324 | 3 | 17.560.976 |
| OTU-96 | 0.3546099 | 0.3546099 | 2 | 16.860.466 |
| OTU-97 | 0.21929824 | 0.21929824 | 2 | 14.254.385 |
| OTU-98 | 0.0 | 0.0 | 5 | 16.728.624 |
| OTU-99 | 0.0 | 0.0 | 5 | 15.816.326 |
| OTU-100 | 0.28105363 | 0.34843206 | 4 | 12.820.514 |
| OTU-101 | 0 | 0 | 1 | 12.820.514 |
| OTU-102 | 0.16119447 | 0.32051283 | 5 | 16.422.766 |
| OTU-103 | 0.4251449 | 0.84388185 | 5 | 13.948.497 |
| OTU-104 | 0.6143162 | 0.9615385 | 4 | 10.096.153 |
| OTU-105 | 0.16340216 | 0.32786885 | 4 | 17.320.261 |
| OTU-106 | 0.44490817 | 0.5008347 | 3 | 16.753.927 |
| OTU-107 | 0.16129032 | 0.16129032 | 2 | 18.649.517 |
| OTU-108 | 0 | 0 | 1 | 75.862.074 |
| OTU-109 | 0.26709402 | 0.48076925 | 4 | 14.018.692 |
| OTU-110 | 0.64102566 | 0.64102566 | 2 | 14.446.227 |
| OTU-111 | 1.031.896 | 20.725.389 | 4 | 75.862.074 |
| OTU-112 | 0 | 0 | 1 | 15.224.359 |
| OTU-113 | 0.64102566 | 0.64102566 | 2 | 13.621.796 |
| OTU-114 | 0 | 0 | 1 | 37.277.148 |
| OTU-115 | 0.0 | 0.0 | 2 | 37.277.148 |
| OTU-116 | 0 | 0 | 1 | 16.448.597 |
| OTU-117 | 0.5502682 | 0.8695652 | 5 | 10.841.424 |
| OTU-118 | 0.16260162 | 0.16260162 | 2 | 10.841.424 |
| OTU-119 | 0.35206 | 0.6289308 | 5 | 1.364.366 |
| OTU-120 | 0.0 | 0.0 | 2 | 14.435.695 |
| OTU-121 | 0.1754386 | 0.1754386 | 2 | 12.5 |
| OTU-122 | 0.5513143 | 0.83752096 | 5 | 11.928.104 |
| OTU-123 | 0.0 | 0.0 | 3 | 62.295.084 |
| OTU-124 | 0.0 | 0.0 | 2 | 62.295.084 |
| OTU-125 | 0 | 0 | 1 | 18.442.623 |
| OTU-126 | 0 | 0 | 1 | 80.065.365 |
| OTU-127 | 0 | 0 | 1 | 80.065.365 |
| OTU-128 | 0.46896863 | 0.96618354 | 5 | 18.910.255 |
| OTU-129 | 0.2909754 | 0.6535948 | 5 | 14.903.847 |
| OTU-130 | 0.0 | 0.0 | 2 | 12.060.302 |
| OTU-131 | 0.0 | 0.0 | 5 | 9.149.278 |
| OTU-132 | 0 | 0 | 1 | 15.337.424 |
| OTU-133 | 0.22257836 | 0.34722224 | 3 | 16.185.898 |
| OTU-134 | 0.1669449 | 0.5008347 | 3 | 9.917.356 |
| OTU-135 | 0 | 0 | 1 | 9.917.356 |
| OTU-136 | 0 | 0 | 1 | 4.111.842 |
| OTU-137 | 0 | 0 | 1 | 4.111.842 |
| OTU-138 | 0.10683761 | 0.16025642 | 3 | 11.378.204 |
| OTU-139 | 0.09615384 | 0.16025642 | 5 | 11.378.204 |
| OTU-140 | 0.35430816 | 0.82644624 | 5 | 17.924.528 |
| OTU-141 | 0.0 | 0.0 | 5 | 16.366.611 |
| OTU-142 | 0.16420361 | 0.16420361 | 2 | 11.055.277 |
| OTU-143 | 0 | 0 | 1 | 1.762.208 |
| OTU-144 | 0 | 0 | 1 | 14.480.874 |
| OTU-145 | 0.064205706 | 0.16103059 | 5 | 17.006.802 |
