# Supplementary material for: DNA barcoding for the assessment of marine and coastal fish diversity from the Coast of Mozambique
Source: PLoS One. 2024 Feb 6;19(2):e0293345. doi: 10.1371/journal.pone.0293345 (PMC10846724; doi:10.1371/journal.pone.0293345)
Supplement: S3 Table — The Distance Summary reports the sequence divergence between barcode sequences at the species, genus, and family level, and contrasts the distribution of within-species divergence to between-species divergence. (PDF) [file pone.0293345.s003.pdf]

Distance Summary Result - MOZFH (401 records selected)

Distance Summary Tables

The distribution of sequence divergence at each taxonomic level is summarized below. Detailed distance tables can be downloaded by clicking on Details button for each rank.

Label	n	Taxa	Comparisons	Min Dist(%)	Mean Dist(%)	Max Dist(%)	SE Dist(%)
Within Species	352	101	504	0.00	0.21	1.51	0.00
Within Genus	166	25	399	3.64	12.53	23.43	0.01
Within Family	246	19	2002	8.27	17.67	25.52	0.00

Distance Summary Graphs

Sequence divergence for all sequences compared at the species and genus levels.

Within Species

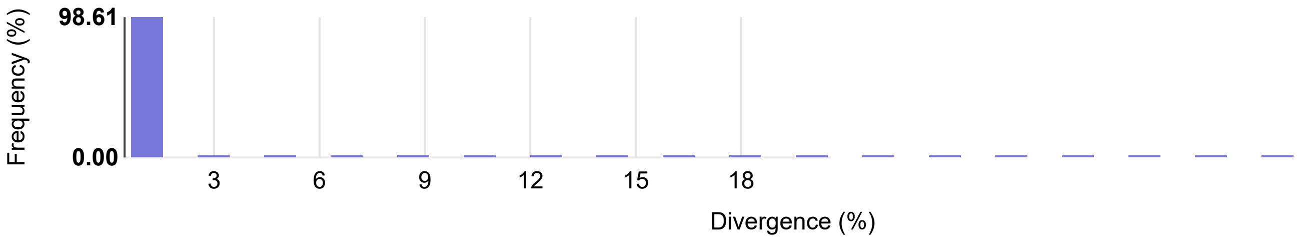

Within Genus

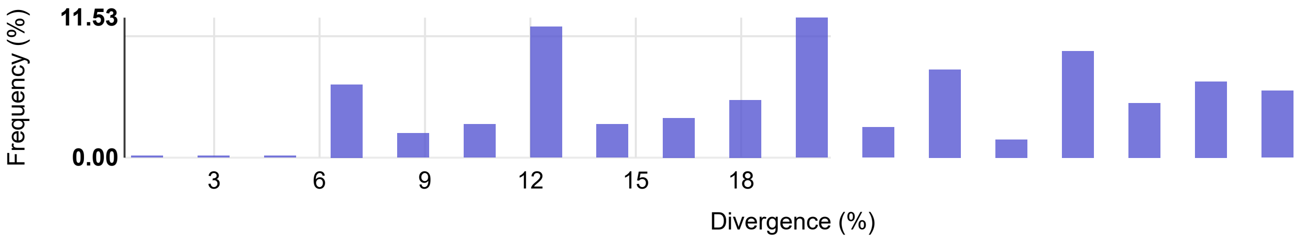

Normalized Results

The within-species distribution is normalized to reduce bias in sampling at the species level. The table below summarizes this distribution, while the histogram plots the distribution of normalized divergence for species (blue) against the genus divergences (red).

Normalized Divergence Statistics

Quantity	Value
Species Count	101
Mean Within-Species Dist (%)	0.23
SE of Mean Within-Species Dist (%)	0.00
Min Between-species Dist (%)	3.64

Normalized Divergence Histogram

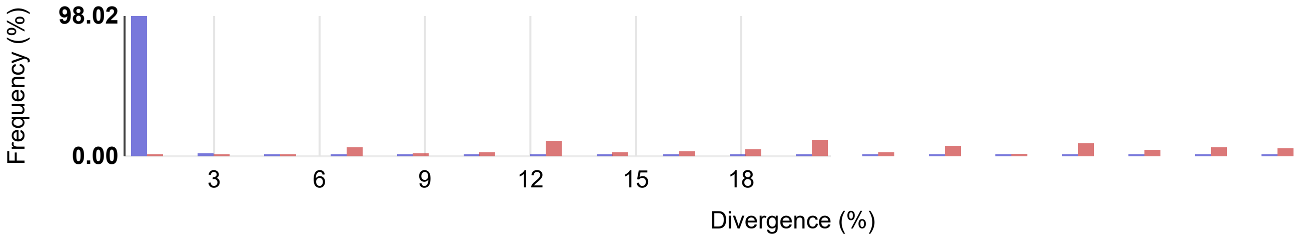

Download Details

Species Genus Family

Analysis description

The Distance Summary reports the sequence divergence between barcode sequences at the species, genus and family level, and also contrasts the distribution of within-species divergence to between-species divergence.

Distance Model: Kimura 2 Parameter
Marker: COI-5P
Deletion Method: Pairwise Deletion
Minimum Complete Columns: 0
Group By: Geography: GPS
Alignment: BOLD Aligner (Amino Acid based HMM)
Filters Applied: ≥ 400bp only

Filter Summary

Data Summary

Copyright BOLD © 2014-2023
