# Supplementary material for: DNA barcoding for the assessment of marine and coastal fish diversity from the Coast of Mozambique
Source: PLoS One. 2024 Feb 6;19(2):e0293345. doi: 10.1371/journal.pone.0293345 (PMC10846724; doi:10.1371/journal.pone.0293345)
Supplement: Attachment — Submitted filename: Comments on the article PONE-D-23-13181.pdf [file pone.0293345.s005.pdf]

Comments on the article PONE-D-23-13181

Title: DNA Barcoding for the Assessment of marine and Coastal Fish Diversity from the Coast of Mozambique"

This paper can be acceptable by revising and addressing the following issues. Need to access the sequences conducted in this study for species identity check. Without accessing the species, I will not confirm the species' accuracy. I want to see the sequences to comment on phylogenetic analysis and species identity.

1. In material and methods, please mention how you can match the valid and accepted species name and families. Authors should follow Eschmeyer's Catalog of Fishes for valid and accepted order, family, and scientific name.
2. The author submits the sequence data into the BOLD database. But did not mention the BIN number in the paper. Please include the BIN number in Table 1. Please be clear about the data availability specifically the sequences, so that reader can find your sequences. I want to see the sequences to comment on phylogenetic analysis and species identity. I encourage the author to submit sequences to the NCBI Gene Bank.
3. Author uses the GBIF database for taxonomic clarification. In the GBIF, you did not find the recent name for all species. So, you should follow Eschmeyer's Catalog of Fishes for valid and accepted order, family, and scientific name
4. In Table 1, Omit the genus column. Please write the species name in the elaborate species column. Use the border in column and row for family range for easy understanding to the reader. Follow the alphabetic order for species of the same family. Please include the Gene Bank accession number in the table for every species. Check the valid and accepted order, family, and scientific name by following Eschmeyer's Catalog of Fishes.
5. In the part of Revealing hidden fish diversity, Write the species name in alphabetic order. such as *Boleophthamus dussumeri, Hippocampus kelloggi, Lethrinus miniatus* and *Maculabatis*
6. Please change Figure 1. Please include a clear picture of the phylogeny to understand the phylogenetic position of species.
7. In the supporting information of Table S1, please follow Eschmeyer's Catalog of Fishes rather than following IUCN for the most updated synonym.
