# Supplementary material for: DNA barcoding for the assessment of marine and coastal fish diversity from the Coast of Mozambique
Source: PLoS One. 2024 Feb 6;19(2):e0293345. doi: 10.1371/journal.pone.0293345 (PMC10846724; doi:10.1371/journal.pone.0293345)
Supplement: Attachment — Submitted filename: Reviewed on PONE-D-23-13181.pdf [file pone.0293345.s006.pdf]

PLOS ONE

DNA barcoding for the assessment of marine and coastal Fish Diversity from the Coast of Mozambique

--Manuscript Draft--

**Manuscript Number:**	PONE-D-23-13181
**Article Type:**	Research Article
**Full Title:**	DNA barcoding for the assessment of marine and coastal Fish Diversity from the Coast of Mozambique
**Short Title:**	DNA barcoding of the Fish species from Mozambique
**Corresponding Author:**	Valdemiro Muhala, PhD candidate<br>Instituto Superior Politecnico de Gaza<br>Chokwé, Gaza MOZAMBIQUE
**Keywords:**	DNA barcoding; Cytochrome C Oxidase I; Diversity; Fish; Mozambique
**Abstract:**	The ichthyological provinces of Mozambique are understudied hotspots of global fish diversity. In this study, we applied DNA barcoding to identify the composition of the fish fauna from the coast of Mozambique. A total of 143 species belonging to 104 genera, 59 families, and 15 orders were identified. The overall K2P distance of the COI sequences within species ranged from 0.00% to 1.51%, while interspecific distances ranged from 3.64% to 24.49%. Moreover, the study revealed 15 threatened species according to the IUCN Red List of Threatened Species, with elasmobranchs being the most represented group. Additionally, the study also uncovered four new species that were not previously recorded in this geographic area, including Boleophthalmus dussumieri, Maculabatis gerrardi, Hippocampus kelloggi, and Lethrinus miniatus. This study represents the first instance of utilizing molecular references to explore the fish fauna along the Mozambican coast. Our results indicate that DNA barcoding is a dependable technique for the identification and delineation of fish species in the waters of Mozambique. The DNA barcoding library established in this research will be an invaluable asset for advancing the understanding of fish diversity and guiding future conservation initiatives.
**Order of Authors:**	Valdemiro Muhala, PhD candidate
	Aurycéia Guimarães-Costa, PhD
	Isadola Macate, Master
	Luan Rabelo, Master
	Adam Bessa-Silva, PhD
	Luciana Watanabe, PhD
	Gisele dos Santos, BSc
	Luísa Sambora, Master
	Marcelo Vallinoto, PhD
	Iracilda Sampaio, PhD
**Additional Information:**	
**Question**	**Response**
**Financial Disclosure**<br><br>Enter a financial disclosure statement that describes the sources of funding for the work included in this submission. Review the submission guidelines for detailed requirements. View published research	This research was financed by Conselho Nacional de Desenvolvimento Científico e Tecnológico (CNPq) through a research project 407536/2021-3, 309916/2021-6 and the APC was funded by Pro-Reitoria de Pesquisa e Pós-Graduação of the Universidade Federal do Pará.

articles from *PLOS ONE* for specific examples.<br><br>This statement is required for submission and **will appear in the published article** if the submission is accepted. Please make sure it is accurate.<br><br>**Unfunded studies**<br>Enter: *The author(s) received no specific funding for this work.*<br><br>**Funded studies**<br>Enter a statement with the following details:<br>• Initials of the authors who received each award<br>• Grant numbers awarded to each author<br>• The full name of each funder<br>• URL of each funder website<br>• Did the sponsors or funders play any role in the study design, data collection and analysis, decision to publish, or preparation of the manuscript?<br>• **NO** - Include this sentence at the end of your statement: *The funders had no role in study design, data collection and analysis, decision to publish, or preparation of the manuscript.*<br>• **YES** - Specify the role(s) played.<br><br>* typeset	
**Competing Interests**<br><br>Use the instructions below to enter a competing interest statement for this submission. On behalf of all authors, disclose any competing interests that could be perceived to bias this work—acknowledging all financial support and any other relevant financial or non-financial competing interests.<br><br>This statement is required for submission and **will appear in the published article** if the submission is accepted. Please make sure it is accurate and that any funding sources listed in your Funding Information later in the submission form are also declared in your Financial Disclosure statement.	The authors declare no conflict of interest.

Powered by Editorial Manager® and ProduXion Manager® from Aries Systems Corporation

View published research articles from *PLOS ONE* for specific examples.<br><br>**NO authors have competing interests**<br><br>Enter: *The authors have declared that no competing interests exist.*<br><br>**Authors with competing interests**<br><br>Enter competing interest details beginning with this statement:<br><br>*I have read the journal's policy and the authors of this manuscript have the following competing interests: [insert competing interests here]*<br><br>* typeset	
**Ethics Statement**<br><br>Enter an ethics statement for this submission. This statement is required if the study involved:<br><br>• Human participants<br>• Human specimens or tissue<br>• Vertebrate animals or cephalopods<br>• Vertebrate embryos or tissues<br>• Field research<br><br>Write "N/A" if the submission does not require an ethics statement.<br><br>General guidance is provided below. Consult the submission guidelines for detailed instructions. **Make sure that all information entered here is included in the Methods section of the manuscript.**	The specimens used in this study were obtained through direct acquisitions from local artisanal fishermen at landing sites and traders in fish markets. No live fish were captured or maintained in captivity for tissue collection purposes, thereby eliminating the requirement for any collection permits.

**Format for specific study types**<br><br>**Human Subject Research (involving human participants and/or tissue)**<br>• Give the name of the institutional review board or ethics committee that approved the study<br>• Include the approval number and/or a statement indicating approval of this research<br>• Indicate the form of consent obtained (written/oral) or the reason that consent was not obtained (e.g. the data were analyzed anonymously)<br><br>**Animal Research (involving vertebrate animals, embryos or tissues)**<br>• Provide the name of the Institutional Animal Care and Use Committee (IACUC) or other relevant ethics board that reviewed the study protocol, and indicate whether they approved this research or granted a formal waiver of ethical approval<br>• Include an approval number if one was obtained<br>• If the study involved *non-human primates*, add *additional details* about animal welfare and steps taken to ameliorate suffering<br>• If anesthesia, euthanasia, or any kind of animal sacrifice is part of the study, include briefly which substances and/or methods were applied<br><br>**Field Research**<br><br>Include the following details if this study involves the collection of plant, animal, or other materials from a natural setting:<br>• Field permit number<br>• Name of the institution or relevant body that granted permission	
**Data Availability**<br><br>Authors are required to make all data underlying the findings described fully available, without restriction, and from the time of publication. PLOS allows rare exceptions to address legal and ethical concerns. See the PLOS Data Policy and FAQ for detailed information.	Yes - all data are fully available without restriction

A Data Availability Statement describing where the data can be found is required at submission. Your answers to this question constitute the Data Availability Statement and **will be published in the article**, if accepted.<br><br>**Important:** Stating 'data available on request from the author' is not sufficient. If your data are only available upon request, select 'No' for the first question and explain your exceptional situation in the text box.<br><br>Do the authors confirm that all data underlying the findings described in their manuscript are fully available without restriction?	
**Describe where the data may be found in full sentences. If you are copying our sample text, replace any instances of XXX with the appropriate details.**<br><br>• If the data are **held or will be held in a public repository**, include URLs, accession numbers or DOIs. If this information will only be available after acceptance, indicate this by ticking the box below. For example: *All XXX files are available from the XXX database (accession number(s) XXX, XXX.).*<br>• If the data are all contained **within the manuscript and/or Supporting Information files**, enter the following: *All relevant data are within the manuscript and its Supporting Information files.*<br>• If neither of these applies but you are able to provide **details of access elsewhere**, with or without limitations, please do so. For example:<br><br>*Data cannot be shared publicly because of [XXX]. Data are available from the XXX Institutional Data Access / Ethics Committee (contact via XXX) for researchers who meet the criteria for access to confidential data.*<br><br>*The data underlying the results presented in the study are available from (include the name of the third party*	All relevant data are within the manuscript and its Supporting Information files.

and contact information or URL).<br>• This text is appropriate if the data are owned by a third party and authors do not have permission to share the data.<br><br>* typeset	
Additional data availability information:	Tick here if the URLs/accession numbers/DOIs will be available only after acceptance of the manuscript for publication so that we can ensure their inclusion before publication.

Powered by Editorial Manager® and ProduXion Manager® from Aries Systems Corporation

Manuscript Click here to access/download;Manuscript;Manuscript.docx

DNA barcoding for the assessment of marine and coastal Fish Diversity from the Coast of Mozambique

Valdemiro Muhala [a, b*]; Aurycéia Guimarães-Costa[a*]; Isadola Eusébio Macate[a,c]; Luan Pinto Rabelo[a]; Adam Rick Bessa-Silva[a]; Luciana Watanabe[a]; Gisele Damasceno dos Santos[a]; Luísa Sambora[d]; Marcelo Vallinoto[a] and Iracilda Sampaio[a]

[a]Laboratório de Evolução, Universidade Federal do Pará. Alameda Leandro Ribeiro, Aldeia. Bragança, Pará, Brazil.

[b]Divisão de Agricultura, Instituto Superior Politécnico de Gaza, P.O. Box 1, Chókwè, Mozambique

[c]Universidade Estadual de Santa Cruz, Departamento de Ciências Agrárias e Ambientais Ilheus, BA, Brazil

[d]Universidade Eduardo Mondlane, Escola Superior de Desenvolvimento Rural, Departamento de Produção Agrária, Vilankulos 1304, Moçambique

***Corresponding author:** *Valdemiro dos Pereira Carlos Muhala*

Email: valdemiro.muhala@ispg.ac.mz; auryceia@yahoo.com.br

Abstract

The ichthyological provinces of Mozambique are understudied hotspots of global fish diversity. In this study, we applied DNA barcoding to identify the composition of the fish fauna from the coast of Mozambique. A total of 143 species belonging to 104 genera, 59 families, and 15 orders were identified. The overall K2P distance of the COI sequences within species ranged from 0.00% to 1.51%, while interspecific distances ranged from 3.64% to 24.49%. Moreover, the study revealed 15 threatened species according to the IUCN Red List of Threatened Species, with elasmobranchs being the most represented group. Additionally, the study also uncovered four new species that were not previously recorded in this geographic area, including *Boleophthalmus dussumieri*, *Maculabatis gerrardi*, *Hippocampus kelloggi*, and *Lethrinus miniatus*. This study represents the first instance of utilizing molecular references to explore the fish fauna along the Mozambican coast. Our results indicate that DNA barcoding is a dependable technique for the

identification and delineation of fish species in the waters of Mozambique. The DNA barcoding library established in this research will be an invaluable asset for advancing the understanding of fish diversity and guiding future conservation initiatives.

Keywords: DNA barcoding; Cytochrome C Oxidase I; Diversity; Fish; Mozambique

1. Introduction

The Mozambique Channel comprises an arm of the Indian Ocean located between the Southeast African countries of Madagascar and Mozambique, stretching 1,600 km along the coast [1]. It is recognized as an important diversity hotspot due to a variety of coastal ecosystems that distinguish it from other Western Indian Ocean (WIO) biogeographic provinces. [2, 3]. These systems provide a range of habitats for both animals and plants, making the region abundant in aquatic species [4,5,6]. The diverse habitats include a large rocky coastline in the north with extensive coral reefs, a wider platform in the central part with river outflows and weaker ocean currents that form sandy banks, estuaries, and deltas, and a southern coast characterized by wide beaches, bays, and seagrass beds with numerous endemic species [7, 8, 9].

Investigations into fish species diversity on the west coast of Africa remain in their early stages, largely due to a scarcity of both traditional taxonomists and those employing molecular tools for taxonomic inference within various fish groups [10]. Accurate classification and identification of fish species are important not only for taxonomists but also for various types of research, including fisheries, natural resource surveys, forensic studies, the discovery of cryptic species, and the identification of species not previously known and their conservation status within a specific biogeographic region. [11, 12, 13, 14, 15]. The impacts of lacking of knowledge on ichthyofauna may have even greater repercussions because research focused on fishing activity can be seriously compromised [16].

Historically, surveys of fish fauna off the coast of Mozambique have primarily relied on traditional methods of identification, which use morphological features and meristic counts to distinguish and define fish species. [17, 18, 19]. However, due to subtle morphological differences, complex evolutionary patterns in certain groups, and the existence of cryptic species, these methodologies may not always overcome the challenges of accurately identifying species, resulting in an underestimation of the true diversity of ichthyofauna [20, 21, 22].

These concerns are particularly significant for international organizations focused on biodiversity conservation, which have traditionally relied on the rigid concept of species. This concept has recently been widely challenged by the more comprehensive and integrated approaches adopted by modern taxonomy for classifying living organisms. [23, 24, 25]. Although there is consensus that species represent the smallest independent evolutionary unit, their recognition remains a subject of debate [26, 27], potentially impacting conservation efforts. Furthermore, morphological identification of fish can prove challenging task if not conducted by specialists, leading to inaccurate identifications [28, 29]. Additionally, previous genetic studies have shown that molecular identification may not align with identifications based on morphological characteristics in some instances [30, 31]. This highlights the importance of carrying out a systematic and comprehensive inventory of ichthyofauna using standardized DNA-based methods.

In response to the limitations of classical taxonomy, DNA barcoding has emerged as a widely adopted research method for accurately identifying species [32, 33, 34]. This technique, which relies on the diversity of the Cytochrome C Oxidase I (COI) gene region within mitochondrial DNA, serves as an alternative to traditional taxonomic approaches. DNA barcoding has proven crucial in enhancing the accuracy of species identification and is especially valuable for differentiating between diverse and poorly understood flora and fauna that necessitate species delimitation [32, 33, 34, 35, 36, 37, 38].

Large-scale investigations of ichthyofaunas across various ecosystems have demonstrated that utilizing the cytochrome oxidase I gene captures a significant proportion of known diversity [40, 41, 42]. However, it has also helped to reveal previously unknown diversity [43, 44, 45, 46]. This method is successful due to the availability of reliable DNA barcode reference libraries in the BOLD Systems (Barcode of Life Data Systems) and GenBank [39]. New DNA barcodes can be analysed with available data to check for potential taxonomic conflicts and improve taxonomic resolution [47].

Considering the remarkable potential of DNA barcoding to identify fish species and considering the still unknown fish assemblage in the coast of Mozambique, the aim of this study is to employ this approach to establish a DNA barcoding reference database for the composition of the fish fauna off the coast of Mozambique. This will have significant implications for our understanding of diversity and could contribute to future conservation efforts for marine species in Mozambique.

2.Material and Methods

2.1 Sampling area and specimen collection

The Mozambican coast is in Eastern Africa (Fig 1), spanning over 2.700 km of coastline along the Indian Ocean, second largest African coast [48, 49]. The coastal region showcases a range of diverse landscapes, including sandy beaches, dunes, forests, mangrove swamps, seagrass, and coral reefs. Three distinct ecological zones can be found along the coast, including the Delagoa Basin in the south, the highly productive and rich Sofala Bank in the central region, and the São Lazaro Bank further north [49, 50].

Sampling was conducted randomly across all coastal provinces of the country from December 2019 to April 2022, considering both dry and rainy seasons (Fig 1). Different fishing gear (trawls, surface, and bottom gillnets) commonly used in the country's artisanal fisheries were used for specimen collection. The collected samples were stored in ice boxes and transported to the sorting site, where a substantial portion of the specimens was photographed, and tissues were removed. The specimens were identified to the lowest taxonomic level based on the nomenclature used by the vendors and using species identification keys specific to South-eastern Africa [51, 52].

Approximately 50 mg of muscle tissue was collected from each specimen and preserved in 1.5 ml Eppendorf-type microtubes filled with 96% ethanol. The samples were stored in a freezer set at -20 ºC. After the initial collection of tissues, the specimens were transported to the Evolution Laboratory at the Institute of Coastal Studies of the Federal University of Para, located in Bragança, Pará, Brazil, for the purpose of generating genetic data.

Fig 1. Map of sample origin areas. All the yellow dots correspond to the total areas collected along the coast. The points marked with A to E are Specific illustrations of the different sampling areas.

2.2 Genomic DNA extraction, PCR amplification, and DNA sequencing.

The extraction of DNA was carried out using the Wizard Genomic DNA Purification Kit (Promega Corporation, Madison, WI, USA) according to the manufacturer's protocol. Total genomic DNA was extracted from muscle or fin tissue of fish specimens. To analyse intra- and inter-variability, a partial COI gene from

mitochondrial DNA was used as the molecular marker. The 5′ end of the COI gene was amplified using two pairs of universal primers for fish, FishF1/FishR1 and FishF2/FishR2 [42], resulting in a maximum of 622 bp fragment. The 5′ region of the COI gene was amplified by Polymerase Chain Reaction (PCR) in a 25 µL reaction mixture that consisted of 4 µL of dNTPs (1.25 mM), 2.5 µL of 10X buffer solution, 1 µL of MgCl2 (25 mM), 0.25 µL of each primer (200 ng/µL), 1-1.5 µL of genomic DNA (100 ng/µL), 1 U of Taq DNA polymerase (5 U/µL), and purified water. The amplification process was as follows: an initial denaturation step at 94°C for 3 minutes, followed by 35 cycles of denaturation at 95°C for 1 minute, annealing at 50°C-58°C for 45 seconds, and extension at 72°C for 45 seconds. The process was concluded with a final extension at 72°C for 5 minutes. The positive reactions were then sequenced using an ABI 3500 automatic sequencer (Applied Biosystems).

2.3 Molecular data analysis.

The final sequences were aligned using ClustalW [53], implemented in GENEIOUS 9.0.5 (https://www.geneious.com), and then translated into amino acids to check for potential stop codons using the MEGA 11 software. All the generated sequences were submitted to BOLD database. The high-quality sequences were compared using the National Canter for Biotechnology Information (NCBI) BLAST search engine and the Barcode of Life Data Systems (BOLD) database. The GBIF (Global Biodiversity Information Facility) repositor was used to compile the taxonomic information of the species. After analysing the sequences, parameters such as the presence of barcode gap, sequence length, GC content, and intra/inter genetic distances within and between families, genera, species, and minimum distances to the nearest neighbour were calculated using tools in the BOLD Systems platform using Kimura's two-parameter model and bootstrap of 1000 replicates [54]. Phylogenetic trees were constructed in MEGA 11 using the NJ method based on the Kimura 2-Parameter model with 1000 bootstrap pseudoreplicates [54, 55] and the online tool Interactive Tree of Life (iTOL) [56] were used to visualize and edit the tree.

Analysis of Barcode Index Numbers (BIN) [57] was conducted for species delimitation, based on uncorrected p-distances, which provide a single BIN for each Operational Taxonomic Unit (OTU) obtained from the COI sequences. The analysis of BINs reveals the maximum intra-specific distance and the minimum inter-specific

distance that overlap and allow for species identification. The aligned sequences, primer pairs, trace files, taxonomic information, and collection data were deposited in the Barcode of Life Data Systems under the project MOZFH [57].

3. Results

3.1 DNA barcoding results.

In this study, we generated a final alignment with 419 sequences of the barcode region of COI gene with a length of 622 bp. Of these, we only used sequences with a length of 400 up to 622 bp for genetic analysis. The data correspond to 143 species, 104 genera, 59 families, 15 orders, and 4 unidentified taxa (Table 1 and S1 Table). All samples were assessed morphologically and then subjected to DNA barcoding evaluation to confirm their identification. There were two classes (Teleost and Elasmobranch) of fish samples, including 392 and 27 respectively, which were acquired from local artisanal fishermen at landing sites and fish markets. For more details of the NJ tree clustering and composition see (S1 Fig).

Table 1. List of the 143 fish species, from Mozambican coast waters, which were DNA barcoded.

Order	Family	Genus	Species	Authorship	Status Conservation (IUCN)
Anguilliformes	Muraenesocidae	*Muraenesox*	*M. bagio*	(Hamilton, 1822)	LC
Beloniformes	Hemiramphidae	*Hemiramphus*	*H. far*	(Forsskål, 1775)	NE
Carcharhiniformes	Carcharhinidae	*Carcharhinus*	*C. leucas*	(Müller & Henle, 1839)	VU
		Loxodon	*L. macrorhinus*	(Müller & Henle, 1839)	NT
	Sphyrnidae	*Sphyrna*	*S. lewini*	(Griffith & Smith, 1834)	CR
Clupeiformes	Chirocentridae	*Chirocentrus*	*C. dorab*	(Forsskål, 1775)	LC
	Clupeidae	*Hilsa*	*H. kelee*	(Cuvier, 1829)	LC
		Sardinella	*S. gibbosa*	(Bleeker, 1849)	LC
			S. jussieu	(Lacepède, 1803)	DD
	Engraulidae	*Thryssa*	*T. vitrirostris*	(Gilchrist & Thompson, 1908)	LC
	Pristigasteridae	*Pellona*	*P. ditchela*	(Valenciennes, 1847)	LC
Cypriniformes	Cyprinidae	*Hypophthalmichthys*	*H. molitrix*	(Valenciennes, 1844)	NT

Gadiformes	Merlucciidae	*Merluccius*	*M. paradoxus*	(Franca, 1960)	NE
Mugiliformes	Mugilidae	*Planiliza*	*P. melinoptera*	(Valenciennes, 1836)	NE
		Chelon	*C. dumerili*	(Steindachner, 1870)	DD
		Crenimugil	*C. buchanani*	(Bleeker, 1853)	NE
		Moolgarda	*Moolgarda sp 1*	(Moolgarda)	-
			Moolgarda sp 2	(Moolgarda)	-
		Planiliza	*P. macrolepis*	(Smith, 1846)	LC
Myliobatiformes	Dasyatidae	*Himantura*	*H. uarnak*	(Gmelin, 1789)	EN
		Maculabatis	*M. gerrardi*	(Gray, 1851)	EN
		Pastinachus	*P. ater*	(Macleay, 1883)	VU
	Myliobatidae	*Rhinoptera*	*R. sp*	(Cuvier, 1829)	-
	Plesiobatidae	*Plesiobatis*	*P. daviesi*	(Wallace, 1967)	LC
Perciformes	Acanthuridae	*Acanthurus*	*A. mata*	(Cuvier, 1829)	LC
		Naso	*N. brevirostris*	(Cuvier, 1829)	LC

Caesionidae	*Caesio*	*C. caerulaurea*	(Lacepède, 1801)	LC
		C. xanthonota	(Bleeker, 1853)	LC
		Caesio sp	(Lacepède, 1801)	-
	Pterocaesio	*P. tile*	(Cuvier, 1830)	LC
	Scyris	*S. indica*	(Cuvier & Valenciennes, 1833)	NE
Carangidae	*Platycaranx*	*P. chrysophrys*	(Kimura, Takeuchi & Yadome, 2022)	NE
		P. malabaricus	(Kimura, Takeuchi & Yadome, 2022)	NE
	Turrum	*T. coeruleopinnatum*	(Whitley, 1932)	NE
		T. fulvoguttatum	(Whitley, 1932)	NE
	Alepes	A. djedaba	(Forsskål, 1775)	LC
	Caranx	*C. ignobilis*	(Forsskål, 1775)	LC
		C. papuensis	(Alleyne & Macleay, 1877)	LC
		C. sexfasciatus	(Quoy & Gaimard, 1825)	LC
	Decapterus	*D. macarellus*	(Cuvier, 1833)	LC

	Megalaspis	*M. cordyla*	(Linnaeus, 1758)	LC
	Parastromateus	*P. niger*	(Bloch, 1795)	LC
	Gnathanodon	*G. speciosus*	(Forsskål, 1775)	LC
	Scomberoides	*S. commersonnianus*	(Lacepède, 1801)	LC
		S. tol	(Cuvier, 1832)	LC
	Selar	*S. crumenophthalmus*	(Bloch, 1793)	LC
Cichlidae	*Oreochromis*	*O. mossambicus*	(Peters, 1852)	VU
		O. niloticus	(Linnaeus, 1758)	LC
		O. aureus	(Steindachner, 1864)	NE
	Tilapia	*Tilapia sp*	(Smith, 1840)	NE
Coryphaenidae	*Coryphaena*	*C. hippurus*	(Linnaeus, 1758)	LC
Dinopercidae	*Dinoperca*	*D. petersi*	(Day, 1875)	NE
Ephippidae	*Platax*	*P. teira*	(Forsskål, 1775)	LC
	Tripterodon	*T. orbis*	(Playfair, 1867)	NE

		G. longirostris	(Lacepède, 1801)	LC
Gerreidae	*Gerres*	*G. oyena*	(Forsskål, 1775)	LC
		G. methueni	(Regan, 1920)	NE
Gobiidae	*Boleophthalmus*	*B. dussumieri*	(Valenciennes, 1837)	LC
	Periophthalmus	*P. argentilineatus*	(Valenciennes, 1837)	LC
	Diagramma	*D. pictum*	(Thunberg, 1792)	NE
		P. gaterinus	(Forsskål, 1775)	LC
	Plectorhinchus	*P. gibbosus*	(Lacepède, 1802)	LC
Haemulidae		*P. flavomaculatus*	(Cuvier, 1830)	NE
		P. commersonnii	(Lacepède, 1801)	LC
	Pomadasys	*P. kaakan*	(Cuvier, 1830)	LC
		P. maculatus	(Bloch, 1793)	LC
Istiophoridae	*Istiompax*	*I. indica*	(Cuvier, 1832)	DD
Labridae	*Cheilinus*	*C. trilobatus*	(Lacepède, 1801)	LC

	Cheilio	*C. inermis*	(Forsskål, 1775)	LC
Leiognathidae	*Leiognathus*	*L. equulus*	(Forsskål, 1775)	LC
		L. nebulosus	(Forsskål, 1775)	LC
Lethrinidae	*Lethrinus*	*L. miniatus*	(Forster, 1801)	LC
		L. borbonicus	(Valenciennes, 1830)	LC
Lobotidae	*Lobotes*	*L. surinamensis*	(Bloch, 1790)	LC
		L. argentimaculatus	(Forsskål, 1775)	LC
		L. bengalensis	(Bloch, 1790)	LC
	Lutjanus	*L. erythropterus*	(Bloch, 1790)	LC
Lutjanidae		*L. fulviflamma*	(Forsskål, 1775)	LC
		L. lutjanus	(Bloch, 1790)	LC
	Aprion	*A. virescens*	(Valenciennes, 1830)	LC
	Pristipomoides	*P. filamentosus*	(Valenciennes, 1830)	LC
Menidae	*Mene*	*M. maculata*	(Bloch & Schneider, 1801)	NE

	Parupeneus	*P. indicus*	(Shaw, 1803)	LC
	Upeneus	*U. vittatus*	(Forsskål, 1775)	LC
Nemipteridae	*Scolopsis*	*S. bimaculata*	(Rüppell, 1828)	LC
Nomeidae	*Cubiceps*	*C. whiteleggii*	(Waite, 1894)	NE
Pempheridae	*Pempheris*	*P. connelli*	(Randall & Victor, 2015)	NE
Polynemidae	*Polydactylus*	*P. malagasyensis*	(Motomura & Iwatsuki, 2001)	NE
Priacanthidae	*Priacanthus*	*P. hamrur*	(Forsskål, 1775)	LC
		P. arenatus	(Cuvier, 1829)	LC
Rachycentridae	*Rachycentron*	*R. canadum*	(Linnaeus, 1766)	LC
Scaridae	*Chlorurus*	*C. atrilunula*	(Randall & Bruce, 1983)	LC
		C. sordidus	(Forsskål, 1775)	LC
	Scarus	*S. psittacus*	(Forsskål, 1775)	LC
		S. quoyi	(Valenciennes, 1840)	LC
Sciaenidae	*Argyrosomus*	*A. japonicus*	(Temminck & Schlegel, 1843)	EN

		A. thorpei	(Smith, 1977)	EN
	Otolithes	*O. ruber*	(Bloch & Schneider, 1801)	LC
	Euthynnus	*E. affinis*	(Cantor, 1849)	LC
	Katsuwonus	*K. pelamis*	(Linnaeus, 1758)	LC
Scombridae	*Rastrelliger*	*R. kanagurta*	(Cuvier, 1816)	DD
	Scomberomorus	*S. plurilineatus*	(Fourmanoir, 1966)	DD
		S. commerson	(Lacepède, 1800)	NT
	Thunnus	*T. albacares*	(Bonnaterre, 1788)	LC
	Variola	*V. louti*	(Forsskål, 1775)	LC
	Cephalopholis	*C. argus*	(Schneider, 1801)	LC
		C. boenak	(Bloch, 1790)	LC
Serranidae	*Epinephelus*	*E. rivulatus*	(Valenciennes, 1830)	LC
		E. coioides	(Hamilton, 1822)	LC
		E. areolatus	(Forsskål, 1775)	LC

		E. macrospilos	(Bloch, 1793)	NE
		E. malabaricus	(Bloch & Schneider, 1801)	LC
		E. merra	(Bloch, 1793)	LC
Siganidae	*Siganus*	*S. stellatus*	(Forsskål, 1775)	LC
		S. sutor	(Valenciennes, 1835)	LC
Sillaginidae	*Sillago*	*S. sihama*	(Forsskål, 1775)	LC
Sparidae	*Chrysoblephus*	*C. puniceus*	(Gilchrist & Thompson, 1908)	LC
	Acanthopagrus	*A. vagus*	(Peters, 1852)	VU
		A. berda	(Forsskål, 1775)	LC
	Crenidens	*C. crenidens*	(Forsskål, 1775)	LC
	Dentex	*D. macrophthalmus*	(Bloch, 1791)	LC
	Lithognathus	*L. mormyrus*	(Linnaeus, 1758)	LC
	Pagellus	*P. natalensis*	(Steindachner, 1903)	LC
	Rhabdosargus	*R. thorpei*	(Smith, 1979)	LC

			R. sarba	(Forsskål, 1775)	LC
	Sphyraenidae	*Sphyraena*	*S. jello*	(Cuvier, 1829)	NE
			S. chrysotaenia	(Klunzinger, 1884)	NE
	Terapontidae	*Terapon*	*T. jarbua*	(Forsskål, 1775)	LC
	Trichiuridae	*Trichiurus*	*Trichiurus sp*	(Linnaeus, 1758)	-
Pleuronectiformes	Citharidae	*Paracitharus*	*P. macrolepis*	(Gilchrist, 1904)	DD
	Cynoglossidae	*Cynoglossus*	*C. attenuatus*	(Gilchrist, 1904)	LC
	Paralichthyidae	*Pseudorhombus*	*P. arsius*	(Hamilton, 1822)	LC
	Psettodidae	*Psettodes*	*P. erumei*	(Bloch & Schneider, 1801)	DD
Rhinopristiformes	Rhinidae	*Rhynchobatus*	*R. djiddensis*	(Forsskål, 1775)	CR
			R. australiae	(Whitley, 1939)	CR
	Rhinobatidae	*Acroteriobatus*	*A. leucospilus*	(Giltay, 1928)	CR
Scombriformes	Ariommatidae	*Ariomma*	*A. indicum*	(Day, 1871)	NE
Scorpaeniformes	Platycephalidae	*Cociella*	*C. heemstrai*	(Knapp, 1996)	LC

		Platycephalus	*P. indicus*	(Linnaeus, 1758)	DD
Siluriformes	Ariidae	*Plicofollis*	*P. polystaphylodon*	(Bleeker, 1846)	NE
	Pangasiidae	*Pangasius*	*P. djambal*	(Bleeker, 1846)	LC
Syngnathiformes	Fistulariidae	*Fistularia*	*F. petimba*	(Lacepède, 1803)	LC
	Syngnathidae	*Hippocampus*	*H. kelloggi*	(Jordan & Snyder, 1901)	VU

The species composition was represented by Carangidae (16 species), Sparidae (10 species), Serranidae (9 species), Haemulidae and Lutjanidae (7 species each), Scombridae and Mugilidae (6 species each). The remaining families were composed of fewer than 5 species (Table 1). The sequences generated in this study were all submitted to the BOLD Systems and the species list details are provided in (S1 Table).

The final analysed database had no insertion, deletion, or stop codon, indicating that it accurately represents the mitochondrial COI fragment. Sequences with poor quality DNA were excluded from the analysis. The nucleotide content showed the following average frequency: G=17.77%, C= 28.74%, A=23.88% and T=29.61% with an average GC content of 46.51%.

The NJ tree included 143 distinct OTUs with high bootstrap support of 70/99 (Fig 2). The specimens belonging to the same species exhibited clustering based on both morphological characteristics and genetic similarities, demonstrating a coherent relationship between the two aspects. No evidence of taxonomic deviation was found in any of the species' group, indicating that the DNA barcoding approach accurately identified the analysed species.

Fig 2. NJ tree illustrating the relationships among Mozambican fish species based on DNA barcode sequences. The NJ tree includes both teleost and elasmobranch fish species, constructed using K2P distances from 400 pb barcode sequences (COI). Bootstrap support values are represented by dots at the nodes. Images credits are given to i-iii, vii, xii - (Pollerspöck, J. & Straube, N. 2022, www.shark-references.com; iv - https://link.springer.com/article/10.1007/s12526-021-01208-6; v - Infofish Austrália; vi – FishBase; viii-https://indiabiodiversity.org/; ix-FishBase; xi - https://fishesofaustralia.net.au; xiii - https://www.fishesoftexas.org/; xiv - https://animaldiversity.org/; xv - https://fishider.org; xvi - https://fishesofaustralia.net.au.

3.2 Genetic distances and Barcoding gap.

The Kimura-2-parameter model (K2P) was used to calculate the genetic distance within and between species and the presence of barcode gap. The overall K2P distance of COI sequences are shown in (Table 2). Intraspecific distances ranged from 0.00% to 1.51% (within Species), while interspecific distances ranged from 3.64% to 23.43% (within Genus), which is greater than the minimum intraspecific distance of 2%, the threshold for the identification of fish species by DNA barcoding [58, 59] (Table 2 and Fig 3 A and B). This distance increased with increasing taxonomic level, with distance within families varying from a minimum of 8.27 to a maximum of 25.52 (within Family). The genetic

distance of all species did not exceed the 2%, with the highest intraspecific distance belonging to *Acanthurus mata* OTU-4, with a genetic distance of 1.51% and a barcode gap. The remaining species had distances less than 1%. The minimum nearest neighbor genetic distance was 3.64% from *Epinephelus coioides* OTU-42 to *Epinephelus malabaricus* OTU-41, and the maximum was detected between *Hippocampus kelloggi* OTU-52 and *Carcharhinus leucas* OTU-19, with a genetic distance of 22.08%. All details regarding the species distance are summarized on the (S 3 Table).

Table 2. Summary of the genetic divergence based on (K2P percentage) at each taxonomic level.

Label	n	Taxa	Comparisons	Min Dist (%)	Mean Dist (%)	Max Dist (%)	SE Dist (%)
Within Species	352	101	504	0.00	0.21	1.51	0.00
Within Genus	166	25	399	3.64	12.53	23.43	0.01
Within Family	246	19	2002	8.27	17.67	25.52	0.00

Fig 3. Barcoding gap: Maximum intraspecific Kimura 2-parameter (K2P) distances (A) and mean interspecific K2P distances (B) recorded in fish species from the coast of Mozambique. The graphs show the overlap of the maximum and mean intra-specific distances with the inter-specific (NN = nearest neighbour) distances.

3.3 Revealing hidden fish diversity.

Four species were unexpectedly detected for the first time in this geographical area, whose presence was not expected, including: *Boleophthamus dussumieri, Maculabatis gerrardi, Hippocampus kelloggi* and *Lethrinus miniatus.* The specie *Boleophthalmus dussumieri* is known to be endemic from the Persian Gulf region down to the Indian region [3], and *Lethrinus miniatus* from the Western Pacific [60]. For *B. dussumieri*, our molecular analysis has shown that there is more than one lineage that has not been discussed previously. On the other hand, the data also showed the presence of the Great Seahorse (*Hippocampus kellogi*) in the Central Province of Mozambique. This specie is listed as Vulnerable in the IUCN Red List, and its occurrence is known only from the Indo-Pacific region of Southeast Asia.

The other significant finding from our study was the presence of one of the most invasive fish species, the silver carp (*Hypophthalmichthys molitrix*), which is native to East Asia. This species is classified as "Near Threatened" and has spread globally,

including in the Limpopo River in southern Mozambique. *Rhinoptera sp* was also recorded in this study. However, the occurrence of this species in Mozambique is not officially listed in the global fisheries database. Our molecular data also allowed us to detect for the first time, the presence of *Pangasius djambal* [61], which is known from Southeast Asia, *Dentex macrophthalmus,* which is distributed from the Mediterranean Sea to the eastern Atlantic [62], and *Fistularia petimba*, which is found in the western Atlantic [63].

3.4 Keystones species and Conservation status.

Sharks, stingrays, and seahorses are the most threatened species in the world due to their importance in commercial trade and use for various purposes, and therefore, the conservation and management of these species must be carefully ensured. In our study, we recorded fourteen endangered species (Table 1, Fig 2). Most of them were classified as elasmobranchs: *Acroteriobatus leucospilus*, Endangered (EN); *Carcharhinus leucas*, Vulnerable (VU); *Himantura uarnak*, Endangered (EN); *Loxodon macrorhinus*, Near Threatened (NT); *Maculabatis gerrardi*, Endangered (EN); *Pastinachus ater,* Vulnerable (VU); *Rhynchobatus djiddensis* and *Rhynchobatus australiae,* Critically Endangered (CR); *Sphyrna lewini,* Critically Endangered (CR); and one Syngnathiformes, *Hippocampus kellogi,* Vulnerable (VU).

Most of the teleost species identified in this study (Perciformes) were classified as Least Concern in the IUCN Red List. However, some of them presented a certain level of threat: *Acanthopagrus vagus*, Vulnerable (VU); *Argyrosomus japonicus,* Endangered (EN), *Argyrosomus thorpei,* Endangered (EN), *Hypophthalmichthys molitrix,* Near Threatened (NT) and *Scomberomorus commerson,* Near Threatened (NT).

4. Discussion

This study has demonstrated the utility of DNA barcoding to supplement morphological identification. These results are particularly important because, while the DNA barcoding method has been applied globally to identify fish species, this study provides, for the first time, an overview of the marine fish diversity in the coastal region of Mozambique. This will make a significant contribution to our understanding of the understudied diversity in the Indo-West Pacific biogeographic region.

This study substantially improves our comprehension of fish species and families inhabiting the Mozambique Coast. The prominent occurrence of species within particular families is attributable to both the availability of specimens in the examined areas and the presence of economically important fish species. For instance, the Labridae family is especially abundant in southern Mozambique [64, 65]. In terms of family representation, this research ranks fourth, identifying 60 distinct families. This number is lower than those reported in previous studies, which recorded 90 and 94 families respectively, all using morphological identification methods [65]. Since morphological identification is prone to errors that can lead to misclassification of species, this study presents the most accurate and reliable assessment of fish diversity along Mozambique's coastline to date.

Accurate DNA barcoding species identification relies on their intra- and interspecific genetic distances. Consequently, closely related species may be readily detected in nearby geographic regions of occurrence. To be considered as species divergence, intraspecific genetic distance values must exceed 2%, which is the established threshold for species delimitation [42]. However, no specific threshold has been defined for interspecific or interfamily genetic distances. In the current study, the mean K2P genetic distances for the barcode region of the COI gene at the species, genera, and family levels are: 0.21±0.00%, 12.57±0.01%, and 17.29±0.00%, respectively (Table 2). The average of K2P genetic distances among species, genera, and families in this study was analogous to findings reported in other studies [66, 32, 67]. However, the minimum interspecific distance observed was substantially greater than that reported in [68, 69, 70] for fish in the China Sea, which can be ascribed to the increased taxonomic diversity of fish species in the present study.

In the present study, six species had low genetic distance from each other and clustered clustered closely and two of them were not identified at specie level. The nearest neighbour of *Rhynchobatus australiae* is *R. djadensis* with 3.72%, *Oreochromis mossambicus* X *O. niloticus* with 3.68%, *Epinephelus malabaricus* X *E. coioides*, *Selar crumenophthalmus* X *Selar sp* and *Caesio caerulaurea* X *Caeseo sp* (Figure 2*).* These species, which we could not identify at species level, suggest that they belong to the same genus. Therefore, it is very likely that the sequence of these two morphospecies belongs to the same genus [71].

The barcode gap method was highly effective in distinguishing and delimiting all possible species. Despite the identification of 143 species represented by their respective operational taxonomic units (OTUs), some of these OTUs had remarkably low

intraspecific distance and were treated as single species, yet were grouped into separate OTUs, such as *Molgarda sp1* (OTU-72), *Molgarda sp2* (OTU-73), and *Selar sp.* (OTU-138), and *Selar crumenophthalmus* (OTU-139) (S2 Table). This scenario arises when there is insufficient data in public databases to match the analysed specimens.

4.1 Revealing hidden fish diversity.

The Indo-West Pacific region is renowned for its high diversity of fish species; however, there have been limited or inadequate studies conducted on the composition of fish fauna in the western Indian Ocean. This lack of information can result in an overestimation or underrepresentation of the biodiversity in this region. As an example, this study provides valuable data on the enigmatic presence of the amphibious fish species *B. dussumieri*, whose distribution was previously known to be restricted to the coast of Iran, across the Arabian Sea, and into the Indian region [72, 73].

This study also reports the presence of the stingray species *Maculabatis gerrardi* for the first time. This species is recognized as a complex of species within the Indo Pacific region, with three distinct lineages distributed along its geographic range [74]. Through the analysis of our COI gene sample and available COI gene sequences from GenBank and the BOLD System, our samples were found to cluster with *M. gerrardi* samples from Kwazulu-Natal (JF493650.1, JF493649.1) and Durban (JF493648.1) in South Africa, and the detection of an additional possible lineage in southern Africa (Muhala et al, in preparation) suggests the need for further taxonomic examination of this group of stingrays

Our data has demonstrated the occurrence of *Hippocampus kelloggi*, which significantly expands the known distribution range of the species, previously reported in the regions of China, India, Indonesia, Japan, Malaysia, Philippines, Thailand, Vietnam, and Tanzania [75]. The study confirms the presence of silver carp *Hypophthalmichthys* molitrix in the Limpopo estuary of southern Mozambique, which is widely regarded as one of the most introduced fish species worldwide [76]. The exact circumstances surrounding its introduction to Mozambique for aquaculture purposes, remain unclear due to the inexistence in the list of produced specie in Aquaculture. It is possible that the presence of this species in the region may be attributed to flood runoff from South Africa [77].

The presence of *Lethrinus miniatus* in the western Indian Ocean is surprising, as it is generally found in the Western Pacific, the Ryukyu Islands, eastern Philippines, northern Australia, and New Caledonia [78]. Previous records of its occurrence outside of this range are misidentifications with another species of the same genus, *Lethrinus olivaceus*. However, our molecular data, when compared with studies conducted by [79, 80, 81], affirms the existence of *Lethrinus miniatus* in Mozambican waters.

Our study also documented the presence of *Fistularia petimba*. This species has been reported in several studies as spreading into new areas, including the archipelago of the Azores, Portugal [82], the Aegean Sea [83], the southern Iberian Peninsula [84], the Mediterranean Sea [85, 86], and the Syrian coast [87]. Notably, *Fistularia petimba* has also been recorded in the Gulf of Carpentaria in Australia [88]. Up until now, there have been no previous confirmed reports of its presence in the waters of Mozambique.

In summary, we provide strong evidence of the effectiveness of using DNA barcoding to accurately discriminate and identify many marine and coastal fish species examined thus far. The COI divergence patterns corresponded with morphologically recognized species; however, in some cases, the molecular data revealed previously undetected genetic divergence within a group and instances of low interspecific variation. The presence of cryptic taxa is relatively common among marine animals, emphasizing the need to consider the possibility of neglected diversity and the occurrence of species complexes. The more comprehensive the barcode library, the more useful it will be for the Barcode of Life Initiative [89]. This study represents a significant contribution towards consolidating DNA barcoding as a global system for identifying life forms and enhancing our understanding of the genetic diversity of Mozambican marine fish.

However, even among experienced taxonomists, consistent application of species names remains a challenge, especially when cryptic diversity is present. This is reflected in the conflicting names applied to specimens within the same BIN by different research groups. As the collection of global data increases, barcodes and BINs will play a vital role in integrating taxonomic feedback and significantly contributing to the standardization of names at the international level. This standardization is crucial for the sustainable management of the world's fisheries.

Acknowledgments

We thank Manuel Carlos Lopes, Arnaldo Rumieque, Eusébio Fiquisse, Lucinda Cuamba, Afonso Muhala, Celia Cassamo, Osvaldo Muhala, Graça Malavi, Iolanda Lameira and Gertrudes Ussene for their contribution in acquiring and systematically identifying part of the samples.

5. Reference

1. Swart, N. C., Lutjeharms, J. R. E., Ridderinkhof, H., & De Ruijter, W. P. M. Observed characteristics of Mozambique Channel eddies. *Journal of Geophysical Research: Oceans*. 2010; *115* (C9). https://doi.org/10.1029/2009JC005875

2. Mwachireya, S., Ndagala, J., Moorgawa, S., Ali, H., Randrianandrasana, J., Kimirei, I., & Bhoyroo, V. Draft ocean acidification paper for Western Indian Ocean Region. 2018.

3. Muhala, V., Chicombo, T. F., Macate, I. E., Guimarães-Costa, A., Gundana, H., Malichocho, C.et al. Climate Change in Fisheries and Aquaculture: Analysis of the Impact Caused by Idai and Kenneth Cyclones in Mozambique. *Frontiers in Sustainable Food Systems*. 2021; *5*. https://doi.org/10.3389/fsufs.2021.714187

4. Hoguane, A. M., & Armando, E. V. The influence of the river runoff in the artisanal fisheries catches in tropical coastal waters–The case of the Zambezi River and the fisheries catches in the northern Sofala Bank, Mozambique. *Revista de Gestão Costeira Integrada-Journal of Integrated Coastal Zone Management*. 2015; *15*(4), 443-451. DOI: 10.5894/rgci600

5. Mackay, F., Santos, J., Munga, C., Hoguane, A., Groeneveld, J., & Kuguru, B. Estuarize-WIO: A socio-ecological assessment of small-scale fisheries in estuaries of the Western Indian Ocean. 2021; doi:10.4314/wiojms.si2021.1.1

6. Hoguane, A. M., da Lucia Cuamba, E., & Gammelsrød, T. Influence of rainfall on tropical coastal artisanal fisheries a case study of Northern Mozambique. *Revista de Gestão Costeira Integrada-Journal of Integrated Coastal Zone Management*. 2012; *12*(4), 477-482.

7. Benkenstein, A. Small-scale fisheries in a modernising economy: opportunities and challenges in Mozambique. 2013.

8. Borsa, P., Durand, J. D., Chen, W. J., Hubert, N., Muths, D., Mou-Tham, G, et al. Comparative phylogeography of the western Indian Ocean reef fauna. *Acta oecologica*. 2016; 72, 72-86. https://doi.org/10.1016/j.actao.2015.10.009

9. O'donnell, J. L., Beldade, R., Mills, S. C., Williams, H. E., & Bernardi, G. Life history, larval dispersal, and connectivity in coral reef fish among the Scattered Islands of the Mozambique Channel. *Coral Reefs*. 2017; 36(1), 223-232. https://doi.org/10.1007/s00338-016-1495-z

10. Stein ED, Martinez MC, Stiles S, Miller PE, Zakharov EV. Is DNA Barcoding Actually Cheaper and Faster than Traditional Morphological Methods: Results from a Survey of Freshwater Bioassessment Efforts in the United States? *PLoS ONE*. 2014; 9(4): e95525. https://doi.org/10.1371/journal.pone.0095525

11. Ardura, A., Planes, S., & Garcia-Vazquez, E. Applications of DNA barcoding to fish landings: authentication and diversity assessment. *ZooKeys*. 2013; (365), 49. doi: 10.3897/zookeys.365.6409

12. Bingpeng X, Heshan L, Zhilan Z, Chunguang W, Yanguo W, JianjunW. DNA barcoding for identification of fish species in the Taiwan Strait. *PLoS ONE*. 2018; 13(6): e0198109. https://doi.org/10.1371/journal.pone.0198109

13. Muhala, V., Guimarães- Costa, A., Macate, I. E., Tembe, S., Mula, Y., Tovela, E.et al. First record of mudskipper *Boleophthalmus dussumieri* (Gobiidae: Oxudercinae) on the coast of Mozambique and evidence of two putative lineages along its known distribution range. *Journal of Fish Biology*.2022; *102*(1), 281-286. https://doi.org/10.1111/jfb.15237

14. Patil, T. S., Jamdade, R. A., Patil, S. M., Govindwar, S. P., & Muley, D. V. DNA barcode-based delineation of freshwater fishes from northern Western Ghats of India, one of the world's biodiversity hotspots. *Biodiversity and Conservation*. 2018); *27*, 3349-3371. https://doi.org/10.1007/s10531-018-1604-0

15. Veneza, I., Silva, R., Freitas, L., Silva, S., Martins, K., Sampaio, I.et al. Molecular authentication of Pargo fillets *Lutjanus purpureus* (Perciformes: Lutjanidae) by DNA barcoding reveals commercial fraud. *Neotropical Ichthyology*. 2018; *16*. https://doi.org/10.1590/1982-0224-20170068

16. IMTIAZ, A., NOR S. A. M., & NAIM, D. M. Progress and potential of DNA barcoding for species identification of fish species. *Biodiversitas Journal of Biological Diversity*. 2017; *18*(4), 1394-1405. https://doi.org/10.13057/biodiv/d180415

17. Ko H-L, Wang Y-T, Chiu T-S, Lee M-A, Leu M-Y, et al. Evaluating the Accuracy of Morphological Identification of Larval Fishes by Applying DNA Barcoding. *PLoS ONE*. 2013; 8(1): e53451. doi: 10.1371/journal.pone.0053451

18. Robertson, W. D., M. H. Schleyer, P. J. Fielding, B. J. Tomalin, L. E. Beckley, S. T. Fennessy, et al. Inshore marine resources and associated opportunities for development of the coast of southern Mozambique: Ponta do Ouro to Cabo de Santa Maria. *South African Association for Marine Biological Research* Unpublished Report N° 130. 1996; 1–51. Durban, SAAMBR.

19. Pereira, M. A. M., E. J. S. Videira & K. G. S. Abrantes. Peixes associados a recifes e zonas litorais do sul de Moçambique. *Jornal de Investigação e Advocacia Ambiental*. 2004; 1 (1): 1-20.

20. Matarese AC, Spies IB, Busby MS, Orr JW. Early larvae of *Zesticelus profundorum* (family Cottidae) identified using DNA barcoding. *Ichthyol Res* 58. 2011); 170–174. https://doi.org/10.1007/s10228-010-0198-7

21. Guimarães-Costa, A., Machado, F. S., Reis-Filho, J. A., Andrade, M., Araújo, R. G., Corrêa, E. M. Ret al. DNA Barcoding for the Assessment of the Taxonomy and Conservation Status of the Fish Bycatch of the Northern Brazilian Shrimp Trawl Fishery. *Frontiers in Marine Science*. 2020; *7*, 566021. https://doi.org/10.3389/fmars.2020.566021

22. Ward RD, Hanner R, Hebert PDN . The campaign to DNA barcode all fishes, FISH-BOL. *J Fish Biol*. 2009; 74, 329–356. https://doi.org/10.1111/j.1095-8649.2008.02080.x

23. Garnett, S. T., & Christidis, L. Taxonomy anarchy hampers conservation. *Nature*.2017; *546*(7656), 25-27. https://doi.org/10.1038/546025a

24. Nehal, N., Choudhary, B., Nagpure, A., & Gupta, R. K. DNA barcoding: A modern age tool for detection of adulteration in food. *Critical reviews in biotechnology*. 2021; *41*(5), 767-791. https://doi.org/10.1080/07388551.2021.1874279

25. Galimberti, A., Sandionigi, A., Bruno, A., Bellati, A., & Casiraghi, M. DNA barcoding in mammals: what's new and where next?. *Hystrix*. 2015; *26*(1), 13. https://doi.org/10.4404/hystrix-26.1-11347

26. De Queiroz K. Species concepts and species delimitation. *Systematic Biology*. 2007;56, 879–886. https://doi.org/10.1080/10635150701701083

27. Sites, J. W., & Marshall, J. C. Delimiting species: a Renaissance issue in systematic biology. *Trends in Ecology & Evolution*. 2003; *18*(9), 462-470. https://doi.org/10.1016/S0169-5347(03)00184-8

28. Chen, W., Hubert, N., Li, Y., Xiang, D., Cai, X., Zhu, S., ... & Li, J. Large-scale DNA barcoding of the subfamily Culterinae (Cypriniformes: Xenocyprididae) in East Asia unveils a geographical scale effect, taxonomic warnings, and cryptic diversity. *Molecular Ecology*. 2022; *31*(14), 3871-3887. https://doi.org/10.1111/mec.16540

29. Carvalho, D. C., Palhares, R. M., Drummond, M. G., & Frigo, T. B. DNA Barcoding identification of commercialized seafood in South Brazil: A governmental regulatory forensic program. *Food control*. 2015; *50*, 784- https://doi.org/10.1016/j.foodcont.2014.10.025

30. Dong, W., Liu, J., Yu, J., Wang, L., & Zhou, S. Highly variable chloroplast markers for evaluating plant phylogeny at low taxonomic levels and for DNA barcoding. *PloSone*.2012; *7*(4),e35071. https://doi.org/10.1371/journal.pone.0035071

31. Yang, L., Mayden, R. L., & He, S. Population genetic structure and geographical differentiation of the Chinese catfish Hemibagrus macropterus (Siluriformes, Bagridae): Evidence for altered drainage patterns. *Molecular Phylogenetics and Evolution*. 2009; *51*(2), 405-411. doi:10.1016/j.ympev.2009.01.004

32. Zhang, J. B., & Hanner, R. DNA barcoding is a useful tool for the identification of marine fishes from Japan. *Biochemical Systematics and Ecology*. 2011; *39*(1), 31-42. https://doi.org/10.1016/j.bse.2010.12.017

33. Hebert PD, Gregory TR. The promise of DNA barcoding for taxonomy. *Syst Biol*. 2005; 54:852–859. https://doi.org/10.1080/10635150500354886

34. Hebert, P. D., Ratnasingham, S., & De Waard, J. R. Barcoding animal life: cytochrome c oxidase subunit 1 divergences among closely related species. *Proceedings of the Royal Society of London. Series B: Biological Sciences*. 2003; *270*(suppl_1), S96-S99. https://doi.org/10.1098/rsbl.2003.0025

35. Smith, M. A., Fisher, B. L., & Hebert, P. D. DNA barcoding for effective biodiversity assessment of a hyperdiverse arthropod group: the ants of

Madagascar. *Philosophical Transactions of the Royal Society B: Biological Sciences*. 2005; *360*(1462), 1825-1834. https://doi.org/10.1098/rstb.2005.1714

36. Smith, M. A., Rodriguez, J. J., Whitfield, J. B., Deans, A. R., Janzen, D. H., Hallwachset, al. Extreme diversity of tropical parasitoid wasps exposed by iterative integration of natural history, DNA barcoding, morphology, and collections. *Proceedings of the National Academy of Sciences*. 2008;*105*(34), 12359-12364. https://doi.org/10.1073/pnas.0805319105

37. Smith, M. A., Wood, D. M., Janzen, D. H., Hallwachs, W., & Hebert, P. D. DNA barcodes affirm that 16 species of apparently generalist tropical parasitoid flies (Diptera, Tachinidae) are not all generalists. *Proceedings of the national academy of sciences*. 2007; *104*(12), 4967-4972. https://doi.org/10.1073/pnas.0700050104

38. Tänzler, R., Sagata, K., Surbakti, S., Balke, M., & Riedel, A. DNA barcoding for community ecology-how to tackle a hyperdiverse, mostly undescribed Melanesian fauna. *PLoSOne*.2012; *7*(1),e28832. https://doi.org/10.1371/journal.pone.0028832

39. Ratnasingham S, Hebert PDN. BOLD: The Barcode of Life Data System (www.barcodinglife.org). *Molecular Ecology Notes*. 2007; 7: 355–364. https://doi.org/10.1111/j.1471-8286.2007.01678.x

40. April, J., Mayden, R. L., Hanner, R. H., & Bernatchez, L. Genetic calibration of species diversity among North America's freshwater fishes. *Proceedings of the National Academy of Sciences*. 2011; *108*(26), 10602-10607. https://doi.org/10.1073/pnas.1016437108

41. Hubert, N., Hanner, R., Holm, E., Mandrak, N. E., Taylor, E., Burridge, M, et al. Identifying Canadian freshwater fishes through DNA barcodes. *PLoS one*. 2008; *3*(6), e2490. https://doi.org/10.1371/journal.pone.0002490

42. Ward, R. D., Zemlak, T. S., Innes, B. H., Last, P. R., & Hebert, P. D. DNA barcoding Australia's fish species. *Philosophical Transactions of the Royal Society B: Biological Sciences*. 2005; *360*(1462), 1847-1857. https://doi.org/10.1098/rstb.2005.1716

43. Pereira, L. H., Pazian, M. F., Hanner, R., Foresti, F., & Oliveira, C. DNA barcoding reveals hidden diversity in the Neotropical freshwater fish *Piabina*

argentea (Characiformes: Characidae) from the Upper Paraná Basin of Brazil. *Mitochondrial Dna*. 2011; *22*(sup1), 87-96. https://doi.org/10.3109/19401736.2011.588213

44. Rock, J., Costa, F. O., Walker, D. I., North, A. W., Hutchinson, W. F., & Carvalho, G. R. DNA barcodes of fish of the Scotia Sea, Antarctica indicate priority groups for taxonomic and systematics focus. *Antarctic Science*. 2008; *20*(3), 253-262. https://doi.org/10.1017/S0954102008001120

45. Winterbottom, R., Hanner, R. H., Burridge, M., & Zur, M. A cornucopia of cryptic species-a DNA barcode analysis of the gobiid fish genus Trimma (Percomorpha,Gobiiformes). *ZooKeys*.2014;(381)79. doi: 10.3897/zookeys.381.6445

46. Guimarães-Costa, A. J., Machado, F. S., Oliveira, R. R., Silva-Costa, V., Andrade, M. C., Giarrizzo,T, et al. Fish diversity of the largest deltaic formation in the Americas-a description of the fish fauna of the Parnaíba Delta using DNA Barcoding. *Scientific Reports*. 2019; *9*(1), 1-8. https://doi.org/10.1038/s41598-019-43930-z

47. Knebelsberger, T., Dunz, A. R., Neumann, D., & Geiger, M. F. Molecular diversity of G ermany's freshwater fishes and lampreys assessed by DNA barcoding. *Molecular ecology resources*. 2015; *15*(3), 562-572. https://doi.org/10.1111/1755-0998.12322

48. Carbone, F., & Accordi, G. The Indian Ocean coast of Somalia. *Marine Pollution Bulletin*. 2000; *41*(1-6), 141-159. https://doi.org/10.1016/S0025-326X(00)00107-7

49. Hoguane, A. M. Perfil diagnóstico da zona costeira de Moçambique. *Revista de Gestão Costeira Integrada-Journal of Integrated Coastal Zone Management*, 2007; *7*(1), 69-82.

50. Leal, M. C., Sá, C., Nordez, S., Brotas, V., & Paula, J. Distribution, and vertical dynamics of planktonic communities at Sofala Bank, Mozambique. *Estuarine, Coastal and Shelf Science*. 2009; *84*(4), 605-616. https://doi.org/10.1016/j.ecss.2009.07.028

51. Fischer, W., Sousa, I. M., Silva, C., De Freitas, A., & Poutiers, J. M. Guia de campo das espécies comerciais marinhas e de águas salobras de Moçambique. (1990).

52. Van der Elst, R., Everett, B., Jiddawi, N., Mwatha, G., Afonso, P. S., & Boulle, D. Fish, fishers, and fisheries of the Western Indian Ocean: their diversity and status. A preliminary assessment. *Philosophical Transactions of the Royal Society A: Mathematical, Physical and Engineering Sciences*. 2005; *363*(1826), 263-284. https://doi.org/10.1098/rsta.2004.1492

53. Thompson, J. D., Higgins, D. G., & Gibson, T. J. CLUSTAL W: improving the sensitivity of progressive multiple sequence alignment through sequence weighting, position-specific gap penalties and weight matrix choice. *Nucleic acids research*. 1994; *22*(22), 4673-4680. https://doi.org/10.1093/nar/22.22.4673

54. Kimura, M. A simple method for estimating evolutionary rates of base substitutions through comparative studies of nucleotide sequences. J. *Mol. Evol*. 1980); 16(2), 111–120 https://doi.org/10.1007/BF01731581

55. Tamura, Koichiro; STECHER, Glen; KUMAR, Sudhir. MEGA11: molecular evolutionary genetics analysis version 11. *Molecular biology and evolution*, 2021; v. 38, n. 7, p. 3022-3027. https://doi.org/10.1093/molbev/msab120

56. Letunic, Ivica; BORK, Peer. Interactive Tree Of Life (iTOL) v5: an online tool for phylogenetic tree display and annotation. *Nucleic acids research*, 2021; v. 49, n. W1, p. W293-W296,. https://doi.org/10.1093/nar/gkab301

57. Ratnasingham, S., & Hebert, P. D. A DNA-based registry for all animal species: the Barcode Index Number (BIN) system. *PloS one*. 2013; *8*(7), e66213. https://doi.org/10.1371/journal.pone.0066213

58. Mabragana, E., Díaz de Astarloa, J. M., Hanner, R., Zhang, J., & Gonzalez Castro, M. DNA barcoding identifies Argentine fishes from marine and brackish waters. *PLoS One*. 2011; *6*(12), e28655. https://doi.org/10.1371/journal.pone.0028655

59. Castro Paz, F. P., Batista, J. D. S., & Porto, J. I. R. DNA barcodes of rosy tetras and allied species (Characiformes: Characidae: Hyphessobrycon) from the Brazilian Amazon basin. *PLoS One*. 2014; *9*(5), e98603. https://doi.org/10.1371/journal.pone.0098603

60. Van Herwerden, L., Aspden, W. J., Newman, S. J., Pegg, G. G., Briskey, L., & Sinclair, W. A comparison of the population genetics of Lethrinus miniatus and

Lutjanus sebae from the east and west coasts of Australia: evidence for panmixia and isolation. *Fisheries Research*. 2009; *100*(2), 148-155. https://doi.org/10.1016/j.fishres.2009.07.003

61. Gustiano, R., Prakoso, V. A., & Ath-thar, M. H. F. Asian catfish genus Pangasius: Diagnosis and distribution. *Indonesian Fisheries Research Journal*. 2018; *24*(2), 99-115. http://dx.doi.org/10.15578/ifrj.24.2.2018.99-115

62. Potts, W. M., Inácio, L. A., Santos, C. V., Richardson, T. J., & Sauer, W. H. Aspects of the biology and fisheries of an economically important sparid Dentex macrophthalmus (Bloch 1791) in the Namibe province, Angola. *African Journal of Marine Science*. 2010; *32*(3), 601-611. https://doi.org/10.2989/1814232X.2010.538160

63. Bañón, R., & Sande, C. First record of the red cornetfish *Fistularia petimba* (Syngnathiformes: Fistularidae) in Galician waters: a northernmost occurrence in the eastern Atlantic. *Journal of Applied Ichthyology*. 2008; *24*(1), 106-107. doi: 10.1111/j.1439-0426.2007.00918.x

64. Almeida, A. J., Amoedo, L., & Saldanha, L. Fish assemblages in the seagrass beds at Inhaca Island (Mozambique): cold season. *Boletim do Museu Municipal do Funchal*. 2001;111-125.

65. Pereira, M. A. Preliminary checklist of reef-associated fishes of Mozambique. *MICOA*. 2000; *Maputo*, 21.

66. Wang, Z. D., Guo, Y. S., Liu, X. M., Fan, Y. B., & Liu, C. W. DNA barcoding South China Sea fishes. *Mitochondrial Dna*. 2012; *23*(5), 405-410. https://doi.org/10.3109/19401736.2012.710204

67. Ribeiro, A. D. O., Caires, R. A., Mariguela, T. C., Pereira, L. H. G., Hanner, R., & Oliveira, C. DNA barcodes identify marine fishes of São Paulo State, Brazil. *Molecular Ecology Resources*. 2012; *12*(6), 1012-1020. https://doi.org/10.1111/1755-0998.12007

68. Hou, G., Chen, W. T., Lu, H. S., Cheng, F., & Xie, S. G. Developing a DNA barcode library for perciform fishes in the South China Sea: Species identification, accuracy, and cryptic diversity. *Molecular ecology resources*. 2018; 18(1), 137-146. https://doi.org/10.1111/1755-0998.12718

69. Knebelsberger, T., Landi, M., Neumann, H., Kloppmann, M., Sell, A. F., Campbell, P. D, et al. A reliable DNA barcode reference library for the

identification of the North European shelf fish fauna. *Molecular ecology resources*. 2014; *14*(5), 1060-1071 https://doi.org/10.1111/1755-0998.12238

70. Wibowo, A., Wahlberg, N., & Vasemägi, A. DNA barcoding of fish larvae reveals uncharacterised biodiversity in tropical peat swamps of New Guinea, Indonesia. *Marine and Freshwater Research*. 2016; *68*(6), 1079-1087. https://doi.org/10.1071/MF16078

71. Ramirez, J. L., Birindelli, J. L., Carvalho, D. C., Affonso, P. R., Venere, P. C., Ortega, H. et al. Revealing hidden diversity of the underestimated neotropical ichthyofauna: DNA barcoding in the recently described genus *Megaleporinus* (Characiformes: Anostomidae). *Frontiers in Genetics*. 2017; 149. https://doi.org/10.3389/fgene.2017.00149

72. Murdy, E. O. A redescription of the gobiid fish *Taenioides purpurascens* (Gobiidae: Amblyopinae) with comments on, and a key to, species in the genus. *Ichthyological Research*. 2018; *65*(4), 454-461 https://doi.org/10.1007/s10228-018-0635-6.

73. Last, P.R.; Naylor, G.J.; Manjaji-Matsumoto, B.M. "A revised classification of the family Dasyatidae (Chondrichthyes: Myliobatiformes) based on new morphological and molecular insights". *Zootaxa*. 2016; **4139** (3): 345–368. doi:10.11646/zootaxa.4139.3.2

74. Bineesh, K. K., Gopalakrishnan, A., Akhilesh, K. V., Sajeela, K. A., Abdussamad, E. M., Pillai, N. G. K, et al. DNA barcoding reveals species composition of sharks and rays in the Indian commercial fishery. *Mitochondrial Dna Part A*. 2017; *28*(4), 458-472. https://doi.org/10.3109/19401736.2015.1137900

75. Harasti, D. Southwards range extension of the great seahorse (*Hippocampus kelloggi* Jordan & Snyder, 1901) in Australia. *Journal of Applied Ichthyology*. 2017; *33*(5), 1018-1020. doi:10.1111/jai.13414

76. Stepien, C. A., Snyder, M. R., & Elz, A. E. Invasion genetics of the silver carp *Hypophthalmichthys molitrix* across North America: Differentiation of fronts, introgression, and eDNA metabarcode detection. *PLoS One*. 2019; *14*(3), e0203012. https://doi.org/10.1371/journal.pone.0203012

77. Schneider, M. F. Ocorrencia das Especies Comerciais Exoticasde Peixe no Rio Limpopo depois das Cheias no Ano 2000. *Bole-tim de Investigacao Florestal (Materia Prima)*. 2003; *3*, 31-36.

78. Carpenter, K. E., & Allen, G. R. Emperor fishes and large-eye breams of the world. *FAO Fisheries Synopsis*. 1989; *9*(125), I.

79. Ward, R. D., & Holmes, B. H. An analysis of nucleotide and amino acid variability in the barcode region of cytochrome c oxidase I (cox1) in fishes. *Molecular Ecology Notes*. 2007; *7*(6), 899-907. https://doi.org/10.1111/j.1471-8286.2007.01886.x

80. Lakra, W. S., Verma, M. S., Goswami, M., Lal, K. K., Mohindra, V., Punia, P, et al. DNA barcoding Indian marine fishes. *Molecular Ecology Resources*. 2011; *11*(1), 60-71. https://doi.org/10.1111/j.1755-0998.2010.02894.x

81. Chen, W. J., & Borsa, P. Diversity, phylogeny, and historical biogeography of large-eye seabreams (Teleostei: Lethrinidae). *Molecular phylogenetics and evolution*. 2020; *151*, 106902. https://doi.org/10.1016/j.ympev.2020.106902

82. Azevedo, J. M. N., Raposeiro, P. M., & Rodrigues, L. First records of *Fistularia petimba* and Diodon eydouxii for the Azores, with notes on the occurrence of three additional species. *Journal of Fish Biology*. 2004; *65*(4), 1180-1184. https://doi.org/10.1111/j.0022-1112.2004.00523.x

83. Cerim, H., YAPICI, S., GÜLŞAHİN, A., Soykan, O., & Bilge, G. The first record of the red Cornetfish (*Fistularia petimba* Lacepède, 1803) in the Aegean Sea. *Düzce Üniversitesi Bilim ve Teknoloji Dergisi*. 2021; *9*(2), 607-615. https://doi.org/10.29130/dubited.756372

84. Cárdenas, S., Berastegui, D. A., & Ortiz, J. First record of *Fistularia petimba* Lacepéde, 1803 (Pisces, Fistulariidae) of the coast of Cadiz (southern Iberian Peninsula) (Primera cita de *Fistularia petimba* Lacepéde, 1803 (Pisces, Fistulariidae) en la costa de Cádiz (sur de la península Ibérica)). *Boletín. Instituto Español de Oceanografía*. 1997; *13*(1), 83-86.

85. Ünlüoğlu, A., Akalın, S., Dal, İ., Tıraşın, E. M., & Aydın, C. M. First record of red cornetfish *Fistularia petimba* (Syngnathiformes: Fistulariidae) from Antalya and Iskenderun bays along Turkish coasts of the Mediterranean Sea. *Journal of Applied Ichthyology*. 2018; *34*(4), 977-980. https://doi.org/10.1111/jai.13715

86. Çiftçi, N., Ayas, D., & DOĞANGÜN, M. New locality record for *Fistularia petimba* Lacepède, 1803 from the North-Eastern Mediterranean Sea (Mersin Bay). *Mediterranean Fisheries and Aquaculture Research*. 2019; *2*(2), 42-48.

87. Hussein, C., Ibrahim, A., & Alshawy, F. First record of Red cornetfish, *Fistularia petimba* Lacepède, 1803 (Actinopterygii: Fistulariidae) from the Syrian coast. *International Journal of Aquatic Biology*. 2019; *7*(3), 175-179.

88. Blaber, S. J. M., Brewer, D. T., & Harris, A. N. Distribution, biomass, and community structure of demersal fishes of the Gulf of Carpentaria, Australia. *Marine and Freshwater Research*. 1994; *45*(3), 375-396. https://doi.org/10.1071/MF9940375

89. Ekrem, T., Willassen, E., & Stur, E. A comprehensive DNA sequence library is essential for identification with DNA barcodes. *Molecular phylogenetics and evolution*. 2007; *43*(2), 530-542. https://doi.org/10.1016/j.ympev.2006.11.021

Supporting information

S1 Table. List of the identified species with detailed taxonomic information.

S2 Table. Cluster Sequence Result. The distribution of sequence divergence at each taxonomic level based on the Pairwise Distance model.

S3 Table. Summary of the distance based on the K2P model. The Distance Summary reports the sequence divergence between barcode sequences at the species, genus, and family level, and contrasts the distribution of within-species divergence to between-species divergence.

S 1 Fig. Phylogenetic tree (NJ) of all studied species.

Figure 1

Click here to access/download;Figure;Fig1.tif

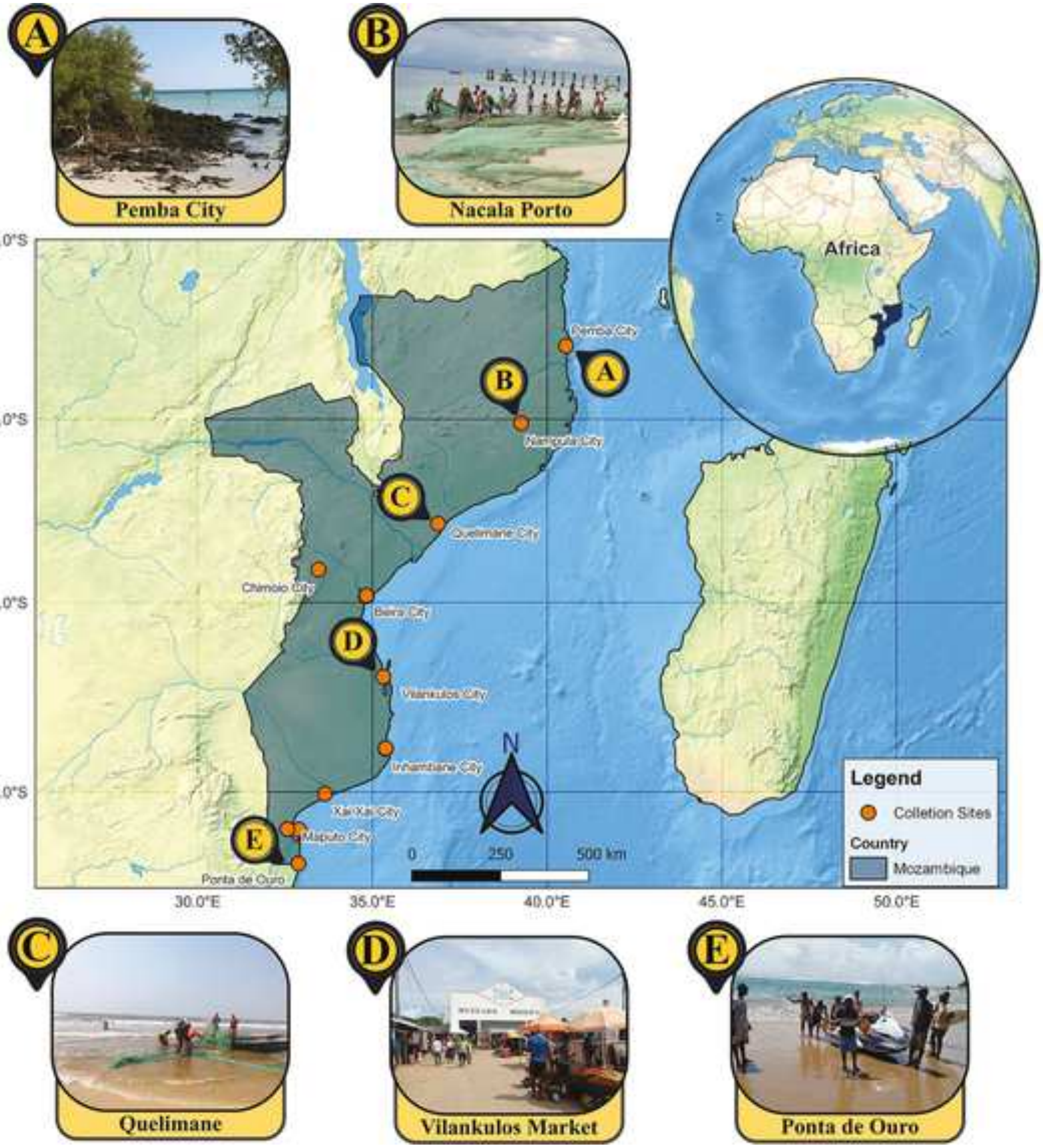

Figure 2 Click here to access/download;Figure;Fig2.tif

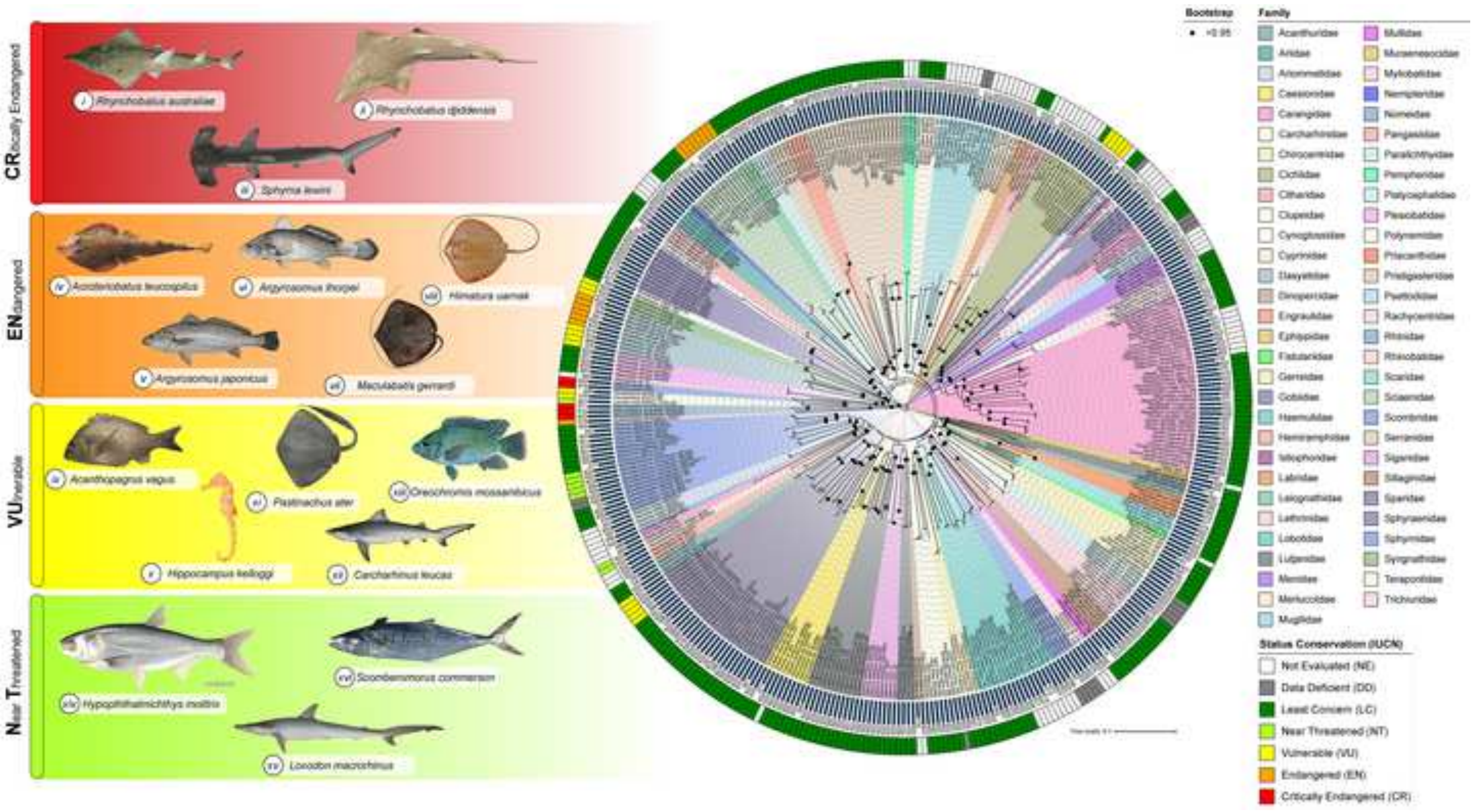

Figure 3

Click here to access/download;Figure;Fig3.tif

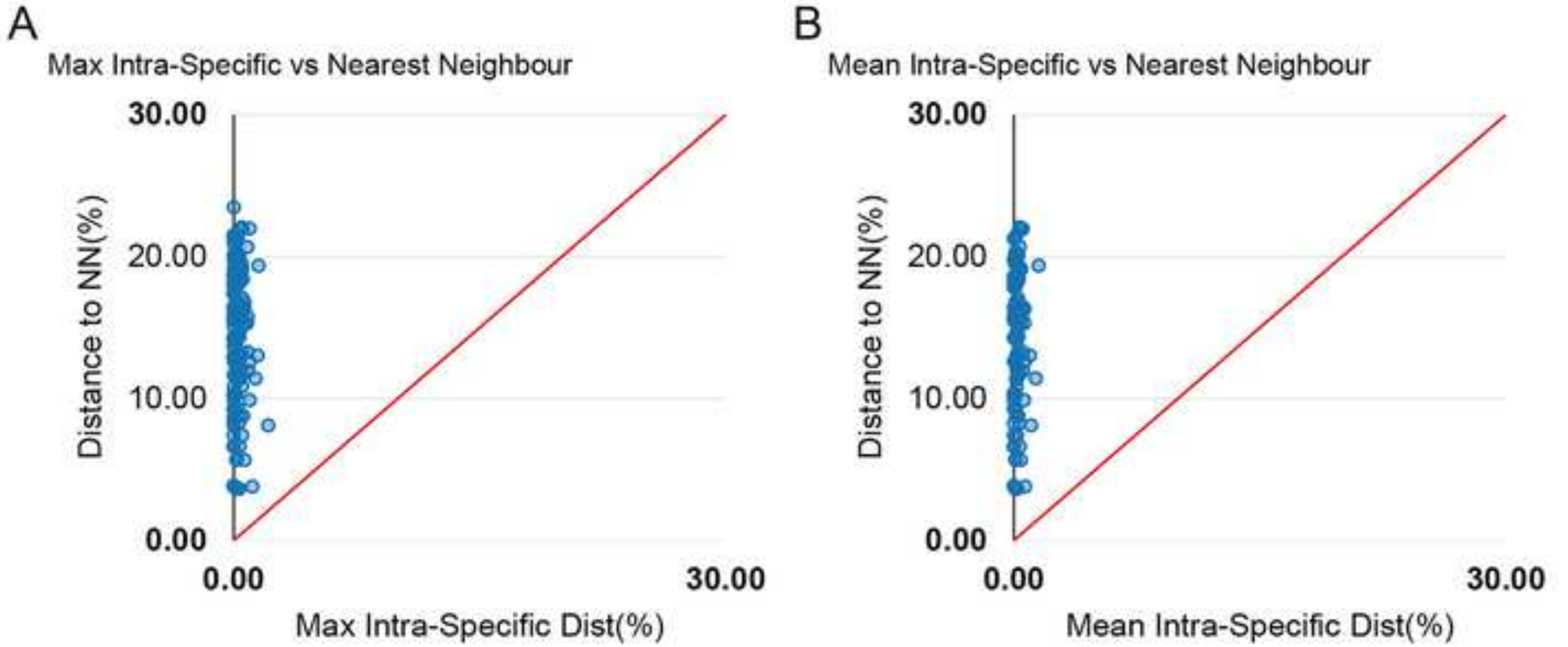

Click here to access/download
Supporting Information
S1 Fig.tif

Click here to access/download
Supporting Information
S1 Table.xlsx

Click here to access/download
Supporting Information
S2 Table.docx

Click here to access/download
Supporting Information
S3 Table.pdf
